# PD-1 protects expanding human T cells from premature restimulation-induced cell death by modulating TCR and CD28 signaling

**DOI:** 10.1038/s41419-026-08530-6

**Published:** 2026-02-26

**Authors:** Katherine P. Lee, Sara Elster, Benjamin Epstein, Camille M. Lake, Andrew L. Snow

**Affiliations:** Department of Pharmacology & Molecular Therapeutics, Uniformed Services University of the Health Sciences, Bethesda, MD USA

**Keywords:** Immune cell death, Cell death and immune response

## Abstract

Programmed cell death-1 (PD-1) is a co-inhibitory receptor expressed on T cells that dampens TCR and CD28 signaling in the immunological synapse. PD-1 is significantly upregulated on T cells in the tumor microenvironment, where it promotes exhaustion in the context of chronic antigen restimulation. Exhaustion renders T cells hyporesponsive and ineffectual, but potentially resistant to restimulation-induced cell death (RICD). Restimulation-induced cell death (RICD) is a critical propriocidal apoptosis program triggered in activated T cells upon robust TCR re-engagement, which serves to constrain effector T cell expansion and longevity to prevent collateral tissue damage. While the checkpoint function of PD-1 has profound implications for cancer immunotherapy, the role of PD-1 in regulating newly activated T cells remains unclear. We hypothesized that PD-1 attenuates RICD sensitivity in human effector T cells by modulating TCR signal strength. Here we show that transient upregulation of PD-1 helps to protect clonally expanding human CD4+ and CD8 + T cells from premature RICD, with only moderate protection noted in terminally-differentiated, PD-1^lo^ effector CD8 + T cells. Restimulation of T cells with beads containing PD-L1 results in significant apoptosis resistance, dependent on PD-L1 dosage and the proximity of PD-L1 to the TCR and CD28. Interestingly, PD-L1 demonstrated a more significant RICD rescue with CD28 co-ligation as opposed to TCR engagement alone, suggesting PD-1 signaling targets both signaling pathways in this context. Furthermore, biochemical/proteomic data suggest PD-1 modulates proximal signaling downstream of both TCR and CD28 and influences the expression of specific pro/anti-apoptotic proteins that govern RICD sensitivity. Despite the original assumption of PD-1 as a programmed death-inducing protein, our research reveals that homeostatic expression of PD-1 in clonally expanding T cells confers RICD resistance that promotes T cell survival and persistence. These findings present significant implications for understanding how blocking or engaging the PD-L1:PD-1 signaling axis may influence apoptosis sensitivity in both normal and exhausted T cells to alter adaptive immune responses.

## Introduction

Adaptive immunity relies on tightly regulated processes to expand and contract populations of effector T cells in response to infection or emerging neoplasms. T cell sensitivity to programmed cell death is carefully and temporally regulated, allowing for the creation of a sufficient effector pool to effectively eradicate a threat (e.g. microbial infection, tumor) while also facilitating removal of surplus cells to leave a durable memory T cell population. Restimulation-induced cell death (RICD) and cytokine withdrawal induced death (CWID) are two apoptotic regulatory pathways that curb the expansion of clonally-specific T cells in the presence of antigen, and induce bulk contraction of T cells after antigen and cytokines wane, respectively [1–3]. RICD is critical for limiting excessive effector T cell production and precluding harmful lymphoproliferation states found in X-linked lymphoproliferative disease 1 (XLP-1) [4], autoimmune lymphoproliferative syndrome (ALPS) [5], and other immune disorders that involve abnormal T cell signaling and apoptotic protein dysregulation [6]. Investigating mechanisms that govern RICD susceptibility will better inform our basic understanding of immune homeostasis and facilitate new therapeutic strategies to reduce immunopathology and enhance engineered immunotherapies by “tuning” effector T cell persistence.

RICD, previously known as AICD (activation-induced cell death) [1, 7, 8], requires strong TCR re-engagement in the presence of IL-2. However, we now appreciate that RICD sensitivity fluctuates over the course of the immune response, and commitment to cell death after restimulation varies by T cell subset, differentiation status, metabolic programs, and other phenotypic/functional states [1–3]. In terminally-differentiated effector T cells, sufficient TCR signal strength is enabled by SLAM-associated protein (SAP) association with the NK, B, and T cell antigen (NTB-A), which enhances proximal TCR signaling to induce pro-apoptotic molecules (e.g., FASL, BIM) and execute RICD [9]. IL-2 is also required for optimal RICD by maintaining cell cycle progression [10–12] and fostering upregulation of FASL, an important driver of RICD in CD4 + T cells but less so in CD8 + T cells [13–16]. Indeed, mice deficient in IL-2 signaling or FAS/FASL function exhibit excessive T cell accumulation and autoimmunity [11, 12]. The relative expression and activity of numerous other proteins also influence effector T cell RICD sensitivity, including DGKα [17], pro-apoptotic proteins such as Nur77/NOR1 [18, 19], BIM [20], perforin/granzymes [21], and Th cytokines such as IFNγ [22], IL-4 [23], and IL-17 [24, 25]. Moreover, anabolic metabolism pathways including glycolysis and fatty acid synthesis appear to sensitize T cells to RICD, in part by regulating expression of these genes [26, 27]. To date, mechanisms responsible for regulating RICD have primarily been studied in mature, terminally-differentiated effector T cells, which are highly sensitive to this pathway. In contrast, clonally expanding T cells are relatively resistant to RICD in spite of abundant IL-2 and antigen, but mechanisms responsible for RICD resistance at this stage remain unclear. Recent work from our lab found that TIM-3, a co-inhibitory protein that modulates TCR signaling cascade, partners with its ligand CEACAM1 to decrease RICD susceptibility in early activated T cells [28]. Earlier work also defined a protective role for CTLA-4 in modulating RICD of Th2 cells [29, 30]. However, the impact of other co-signaling proteins in modulating RICD sensitivity during clonal expansion remains to be investigated.

Co-inhibitory molecules have emerged as prime targets in the emerging field of checkpoint blockade for cancer immunotherapy [31]. In the tumor microenvironment, exhausted, hyporesponsive effector T cells upregulate several co-inhibitory receptors (e.g. PD-1, TIM-3, LAG-3, CTLA-4) in response to chronic antigen stimulation [32, 33]. Blockade of these receptors relieves suppression of T cell signaling and enhances cytotoxic effector function against cancer cells. PD-1 is a hallmark checkpoint inhibitor target, with numerous approved therapeutics blocking PD-1 or its ligand PD-L1 [34]. Targeting PD-1 and/or CTLA-4 has shown remarkable clinical efficacy for some patients harboring specific tumor types, but suboptimal response rates and immune-related adverse events (irAEs) affecting up to 60% of patients can result in treatment failure or cessation [35]. Given the physiologic function of PD-1 in fine-tuning T cell differentiation, metabolism, and effector functions [36], we expect that PD-1 blockade may also dysregulate normal immune responses. However, a role for PD-1 in RICD modulation has not been explored, underscoring our incomplete understanding of normal co-inhibitory receptor function. Given the function of TIM-3 and CTLA-4 in enforcing RICD resistance, we hypothesized that PD-1 decreases RICD-sensitivity by restraining excessive TCR signaling in clonally expanding T cells. Here, we show that PD-1 engagement reduces RICD in human effector T cells by modulating both TCR and CD28 signaling and altering the balance of pro- and anti-apoptotic protein expression to promote RICD resistance.

## Materials and methods

### Human T cell isolation and culture

Peripheral blood mononuclear cells (PBMC) were isolated from de-identified healthy human buffy coats (kindly provided by the NIH Blood Bank) using Ficoll gradient centrifugation. All NIH Blood Bank donors provide informed consent to allow for use of blood products in research (IRB# 99-CC-0168). CD4+ and CD8 + T cells were purified by negative selection using immunomagnetic beads according to the manufacturer’s instructions (StemCell Technologies, kits #17952, 17953). T cells were then activated using anti-CD3/CD28-coupled beads in complete RPMI media (RPMI 1640 (ThermoFisher) + 10% fetal bovine serum (Millipore Sigma) + 1% penicillin/streptomycin (ThermoFisher)) at 37 ^o^C. After 3 days of bead stimulation, activated T cells were thoroughly washed in PBS and cultured in complete RPMI + 100U/ml recombinant IL-2 (Peprotech) to expand effector T cells over several days for use in subsequent experiments.

### Stimulation bead creation

Stimulation beads were created using the Dynabeads™ M-450 Epoxy kit (ThermoFisher, #14011) according to the manufacturer’s protocol. In brief, beads were resuspended and washed with 0.1 M sodium phosphate buffer, pH 7.6. Antibody/protein and Dynabeads were incubated at a ratio of 200 μg protein: 1 mL beads overnight in a 360^o^ rotator-shaker in 1 mL 0.1 M sodium phosphate buffer, pH 7.6, minus the volume of antibody/protein. 0.05% w/v BSA in PBS was added to the protein/bead mixture 15 min after the incubation period started. Beads were subsequently washed 3x with PBS + 0.1% BSA + 2 mM EDTA, pH 7.4, and resuspended in this buffer for long-term storage in the same volume of beads originally allocated. Antibodies and proteins used for bead formulations include anti-CD3 mAb (clone: HIT3α, BD Biosciences, #555336), anti-CD28 mAb (clone: CD28.2, BD Biosciences, #555725), IgG1κ (BioXCell, #BP0297), and recombinant human PD-L1 protein (Biotechne, #156-B7). Bead formulations are described in the Supplemental Table 1.

### RICD assays: purified effector T cells

RICD assays on effector T cells were performed as described in our recent *Bio-protocol* paper [37], with further modulations detailed here. Briefly, RICD was induced in effector T cells using either soluble antibodies or antibody-coupled beads in 96-well round bottom plates (1 × 10^5^ cells/200 μl/well) in triplicate. Soluble anti-CD3 mAb (clone: OKT3, Biogems, #05121-20) was administered in complete RPMI + rIL-2 to a final concentration of 100 ng/mL. For bead stimulations, beads were added to cells at a 1 bead:1 cell ratio. After 24 h restimulation, 1μg/ml propidium iodide (PI, Millipore Sigma) was added to each well; cells were harvested in mini-FACS tubes and quantified on an Accuri C6+ flow cytometer (BD Biosciences) for constant time (15–30 s per sample). Focusing on PI- gated live cells (see Supplementary Fig. 1), % cell loss was calculated using the following formula: (1-(average of triplicate live events plus restim / average of triplicate live events without restim)) x 100. Blocking antibodies including anti-PD-1 mAb (clone: EH12.2H7, Biolegend, #329926; clone: Pembrolizumab, Selleck, #A2005), anti-PD-L1 mAb (clone: Atezolizumab, BioXCell, #SIM0009), and anti-FASL (clone: NOK-1, Biolegend, 306416) were added to cultures 1 hr prior to TCR restimulation at a final concentration of 10 μg/mL. Isotype control antibodies were paired with the corresponding blocking antibody at 10 μg/mL: IgG1κ (BioXCell, #BP0297) or IgG4 (BioXCell, #CP148).

### RICD assay using irradiated PBMC as accessory cells

Activated CD8 + T cells were cultured to day 4 in complete RPMI + rIL-2 as previously described [28]. On Day 3, autologous PBMCs were thawed and rested overnight in complete RPMI. On Day 4, PBMCs were centrifuged for 5 min at 1400 rpm and resuspended at 2 × 10^7^ cells/mL in PBS. PBMCs were stained with a 1 μM solution of carboxyfluorescein succinimidyl ester (CFSE) (Sigma, #21888-25MG-F) for 10 min at 37 °C, followed by immediately adding a 1:1 volume of FBS and then 3 washes in PBS. A Cobalt gamma irradiator (3000 rad) was used to irradiate CFSE-stained PBMCs resuspended in complete RPMI (2 × 10^6^ cells/mL). PBMCs were placed on ice until the T cell RICD plate was prepared as previously described [38]. Briefly, a 96 well plate was prepared for each donor to test PBMC-restimulated RICD in duplicate. T cells (2 × 10^5^) were added to each well with 4 × 10^5^ of autologous, irradiated PBMCs (1:2 ratio) in complete RPMI + 100 U/mL IL-2. Each well was incubated with either anti-PD-1 or anti-PD-L1 blocking Abs or control IgG1κ at 10 μg/mL for 1 hr, after which media (no restimulation), anti-CD3 (clone: OKT3) or anti-CD3 plus anti-CD28 (clone: 28.2) agonistic Abs were added at final concentration of 1 μg/mL. RICD-induced cell loss between unstimulated and restimulated wells was calculated as above by quantifying the difference in live CFSE-negative effector T cells counted by flow cytometry between treatment conditions.

### Flow cytometry for cell surface markers

Cell surface antibody staining was performed on 2–4 × 10^5^ cells per condition with one unstained/isotype control. Briefly, T cells were counted and spun down in polypropylene tubes at 1400 RPM for 5 min. Cells were washed 3x in 2 mL FACS buffer (1% FBS and 0.2% NaN_3_ in PBS) at 1400 RPM for 5 min. Cells were then resuspended in 100 μL FACS buffer minus the antibody volume (typically 5 μL of florescent antibody used at this concentration of cells). Samples were stained on ice in the dark for at least 30 min, washed 3x and resuspended at 1 × 10^6^ cells/mL in FACS buffer for flow cytometric analysis. Antibodies used for surface staining are included in Supplemental Table 2. Propidium iodide (final concentration = 2 μg/mL) and FSC/SSC plots were used to differentiate live and dead cells. Cells were analyzed using an Accuri C6+ flow cytometer (Becton Dickinson). Flow cytometric data were analyzed using FlowJo version 10.8.1.

### Annexin V and propidium iodide (PI) staining

Day 4 and Day 11 effector T cells (2 × 10^5^ cells/well), were restimulated as described above with anti-CD3/CD28/IgG or anti-CD3/CD28/PD-L1-Fc beads for 6 hr in a 96 well plate. Cells were collected and washed twice in polypropylene tubes with 2 mL FACS buffer. Cells were then stained with 1.5 μl of Annexin V APC (Biolegend, #640920) in 200 μL of Annexin V Binding Buffer (Biolegend, #422201) for 15 min at room temperature (RT) in the dark. After incubation, 10 μL of 40 μg/mL propidium iodide was added to each sample and then immediately collected on an Accuri C6+ flow cytometer (ungated).

### PI cell cycle analysis

Day 4 and Day 11 effector T cells were restimulated as above with the applicable antibody or bead for 18 hr. Cells were collected, washed 1x in PBS, and then stained on ice for 30 min with 0.5 mL of freshly prepared propidium iodide staining solution (50 μg/mL propidium iodide (Thermo Fisher, P1304MP), 0.1% Triton X-100 (Sigma, #648463-50 ML), 1 mg/mL sodium citrate (Sigma, W302600-1KG-K), and 1 mg/mL RNAse A (Sigma, #10109142001) in sterile water). Cells were immediately analyzed by flow cytometry, reading PI fluorescence on a linear scale. Gating and analysis of cell cycle stage distribution was performed in FlowJo.

### pERK intracellular flow cytometry

Intracellular flow cytometry experiments were performed on Day 4 effector T cells (2.5 × 10^5^ cells per condition) cultured without IL-2 for 24 hr. Cells were then starved in 2% BSA-PBS solution at 37 ^o^C for 2 hr in non-tissue culture treated plate. After starvation, cells were plated and left unstimulated or stimulated with beads at 1:1 ratio. The plate was spun down at 1400 RPM for 5 min and incubated at 37 °C. At each time point, cells were removed from the plate and transferred to polypropylene tubes filled with ice cold PBS to curtail signaling. Tubes were spun down at 1400 RPM for 5 min and fixed with a 1.5% PFA solution in PBS at RT for 10 min. Cells were washed 1x with cold FACS buffer, and then 400 μL of ice-cold 100% methanol was added dropwise while vortexing to permeabilize cells. Samples were incubated on ice for 20 min and then washed again 1x with cold FACS buffer. Fixed/permeabilized cells were given 2 μL of either a control isotype antibody (Rabbit mAb IgG XP® Isotype Control, Clone: DA1E, Cell Signaling, #2985S) or Phospho-p44/42 MAPK (Erk1/2) Alexa Fluor® 647 mAb (Clone: 197G2, Cell Signaling, #13148S) in 98 μL of FACS buffer and incubated for 30 min on ice. Samples were washed once more and resuspended in 200 μL cold FACS buffer for flow cytometric analysis.

### Immunoblotting

Day 4 T cells were stimulated for the desired time based on experimental conditions. For phosphoprotein analysis, cells were cultured without IL-2 for 24 hr, starved in 2% BSA-PBS mixture 2 hr prior to restimulation, and spun down on a plate with beads (1:1) at 1400 RPM for 5 min prior to lysis and protein processing. After restimulation, cells were washed 3x with ice cold PBS and resuspended in 12 μL RIPA buffer (0.137 M NaCl, 20 mM Tris pH 8.0, 10% glycerol, 1% NP-40 0.1% SDS, 0.1% sodium deoxycholate, protease inhibitors (Sigma, #11836153001), and phosphatase inhibitors (Sigma, #4906845001)) per 1 million cells. Cells were lysed and incubated on ice for 1 hr, vortexing for 10 sec every 15 min. After incubation, lysates were spun down at 12,800 RPM for 10 min in a centrifuge at 4 ^o^C to remove insoluble material. Lysates from each condition were combined 1:1 with 2x Laemmli Sample Buffer (Bio-Rad, #1610737) supplemented with 5% β-mercaptoethanol (Sigma, #M6250-10ML), and then denatured for 5 min at 95 ^o^C. Protein aliquots were separated by SDS-PAGE using 4–20% precast polyacrylamide gels (Bio-Rad, #4561095) and subsequently transferred to nitrocellulose for 10 min using a Trans-Blot Turbo system (Bio-Rad). Membranes were blocked with Intercept Blocking Buffer (Bio-Rad, #927-70001) for 30 min, rocking at RT. Primary antibodies were diluted in blocking buffer according to manufacturer’s recommendations and incubated with membranes overnight at 4 ^o^C. A list of immunoblotting antibodies is provided in Supplementary Table 2. Secondary antibodies were used at a 1:10,000 dilution from LI-COR (listed in the Materials Table) and imaged on the Odyssey DLx (LI-COR). Images were analyzed and quantified using the Image Studio™ software (LI-COR).

### Proteome profiler human apoptosis array

Day 4 effector T cells derived from 4 different human donors were plated individually at 1.4 × 10^6^ cells/mL in complete RPMI with IL-2 and treated as follows: unstimulated, p328-IgG (1:1), p328-L1 bead (1:1), or staurosporine (1 μM) (Cayman Chemical, #81590). The plate was spun at 1400 RPM for 5 min and placed back in the incubator for 5 hr. After stimulating, 2 × 10^6^ cells from each well were lysed in 25 μL of RIPA buffer. Remaining cells (5 × 10^6^) for each condition were washed with PBS and lysed in 200 μL of Lysis Buffer 17 supplemented with a protease inhibitor cocktail (Bimake, #B14001). Lysates were gently rocked for 30 min at 4 ^o^C, then centrifuged at 12,800 RPM for 5 min. BCA protein quantification (Thermo Fisher, #23227) was performed to standardize each stimulation condition and donor. Each array [4] was incubated overnight at 4 ^o^C with 300 μg of total protein, representing pooled lysates (75 μg per donor) from each stimulation condition, and processed further according to manufacturer instructions. Arrays were imaged on the Azure c400 imaging system (Azure Biosystems) and exposed for 8 min. Protein intensity was quantified using Quick Spots software (Ideal Systems).

### Statistics

All statistics were performed using GraphPad Prism 9 software (Dotmatics). Two-sided statistical analyses were used in all experiments. Paired student *t* tests were used in human donor associated experiments based on normal distribution. Paired donor data is represented by connected lines in figures. For the PD-L1 bead titration experiment, a two-way ANOVA was utilized with matched values. *P* values were quantified using the following nomenclature: ns = *p* > 0.05, * = *p* ≤ 0.05, ** = *p* ≤ 0.01, *** = *p* ≤ 0.001, and **** = *p* ≤ 0.0001. Error bars used represent standard deviation across donors of a particular experimental condition while bars represent sample mean. Sample size (N) represents the number of individual biological replicates for each experiment. All experiments were replicated at least 3 times using T cells derived from distinct human donors, including 3 technical replicates for each experiment.

## Results

### PD-1 engagement reduces RICD in human effector T cells

**Fig. 1:**
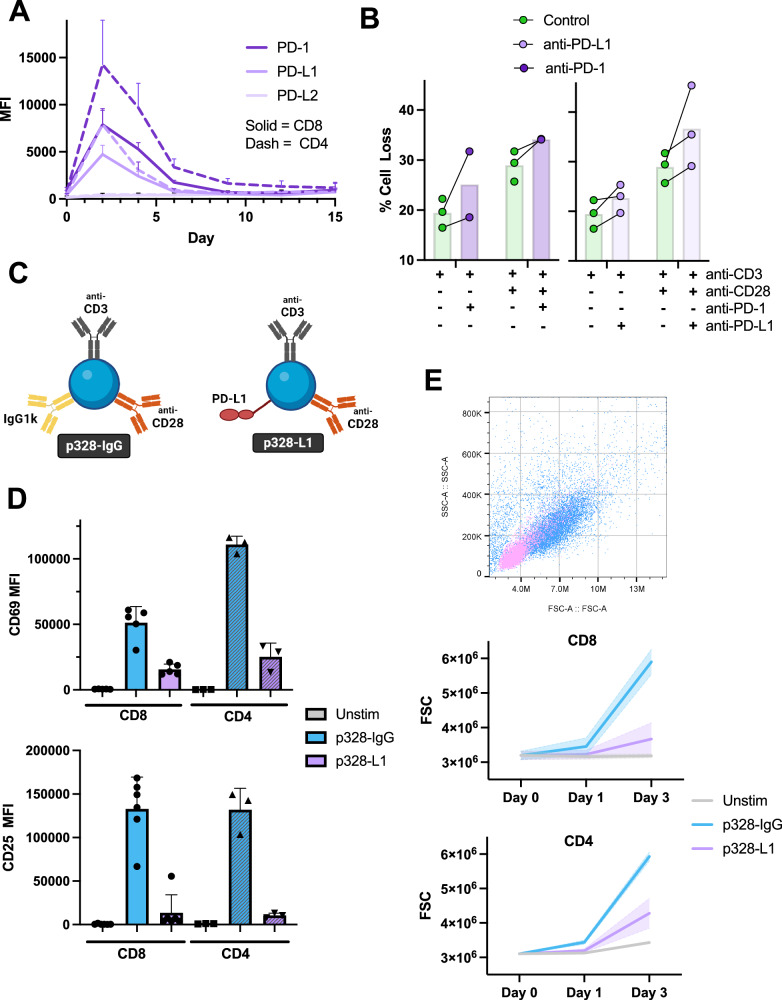
PD-1 is highly expressed in clonally expanding T cells and impacts T cell activation and survival. T cells isolated from healthy human donors were activated with anti-CD3/28 beads and IL-2 was added on Day 3. **A** PD-1, PD-L1, and PD-L2 expression were measured via flow cytometry over 15 days for *N* = 8 in CD4+ and *N* = 5 in CD8 + T cells. **B** Day 4 purified CD8 + T cells were restimulated with autologous irradiated PBMC and anti-CD3 (1 μg/mL) ± anti-CD28 (1 μg/mL) in the presence of control antibody, anti-PD-1, or anti-PD-L1 at 10 μg/mL for 20 hr. Death was assessed via flow cytometry by propidium iodide staining. Single dot correlates with one human donor. Connecting line compares change in untreated and treated samples within the same donor. *N* = 3. **C** Diagram illustrating the proxy APC (pAPC) design created with a 1:1:1 ratio of anti-CD3, anti-CD28, and IgG1κ or recombinant human Fc-chimera PD-L1. **D** Freshly isolated CD4+ or CD8+ human T cells were activated at a 1:1 cell:bead ratio with IgG or PD-L1 beads and monitored via flow cytometry for CD69 expression after 24 hr (*N* = 3 CD4 + , *N* = 5 CD8 + ) and CD25 expression after 72 hr (*N* = 3 CD4 + , *N* = 6 CD8 + ). **E** Blastogenesis patterns of unactivated, IgG, or PD-L1 activated cells on Day 3 are demonstrated by dot plots assessing forward scatter (FSC) (x-axis) and side scatter (SSC) (y-axis). The progressive changes in FSC monitored via flow cytometry over 3 days are summarized for CD4+ and CD8 + T cells. *N* = 3 CD4 + , *N* = 5 CD8 + .

PD-1 is rapidly expressed upon T cell activation and in response to subsequent TCR restimulation, especially in the context of chronic antigen exposure [39, 40]. To characterize the expression of PD-1 and relevant ligands in our primary human T cell model, we purified CD4+ and CD8 + T cells separately and activated them in vitro with anti-CD3/anti-CD28-ligated beads while monitoring PD-1, PD-L1, and PD-L2 expression over the course of 15 days (Fig. 1A). PD-L1 and PD-1 were rapidly expressed upon T cell activation, peaking around day 2 before declining to reach a low level of stable expression after one week of in vitro culture. Expression of PD-1 and PD-L1 during peak clonal expansion were ~10 and 18 times higher than baseline for CD8 + T cells and CD4 + T cells, respectively, whereas PD-L2 expression remained negligible [33]. Interestingly, CD4 + T cells expressed twice the amount of PD-1 as CD8 + T cells in our system. To test the functional significance of PD-1/PD-L1 expression and signaling during clonal expansion, we first co-cultured day 4 T cells with anti-CD3/anti-CD28 Abs in the presence of autologous irradiated, CFSE-labeled PBMC to act as accessory cells for antibody crosslinking and PD-L1/L2 expression (Fig. 1B). RICD was readily observed in co-cultured, CFSE^-^ T cells. Concomitant blockade of PD-1:PD-L1 signaling using pembrolizumab (anti-PD-1) or atezolizumab (anti-PD-L1) resulted in a modest but significant increase in cell death, supporting our hypothesis that PD-1 signaling can confer protection against RICD. In contrast, blocking PD-1-PD-L1 interactions in purified T cell cultures had a minimal impact on RICD (Supplementary Fig. 2), implying PD-1:PD-L1 interactions between adjacent T cells do not influence TCR restimulation.

To enforce PD-1 signaling during RICD, we ligated beads with anti-CD3, anti-CD28, and either an isotype antibody (p328-IgG) or recombinant human PD-L1-Fc protein (p328-L1) (Fig. 1C) to use as a “proxy antigen presenting cells (pAPC)” for T cell restimulation. We first evaluated their effect on resting T cell activation. Similar to prior studies [41], both CD69 and CD25 upregulation were substantially reduced in CD4+ and CD8 + T cells stimulated with p328-L1 beads relative to p328-IgG control beads (Fig. 1D). Decreased proliferation and blastogenesis (reduced forward scatter on flow cytometry) were also observed with p328-L1 beads (Fig. 1E), confirming that enforced PD-1 signaling can markedly dampen T cell priming.

### PD-1 induces dose-dependent RICD rescue in CD4+ and CD8 + T cells by functioning in the “immunological synapse”

**Fig. 2:**
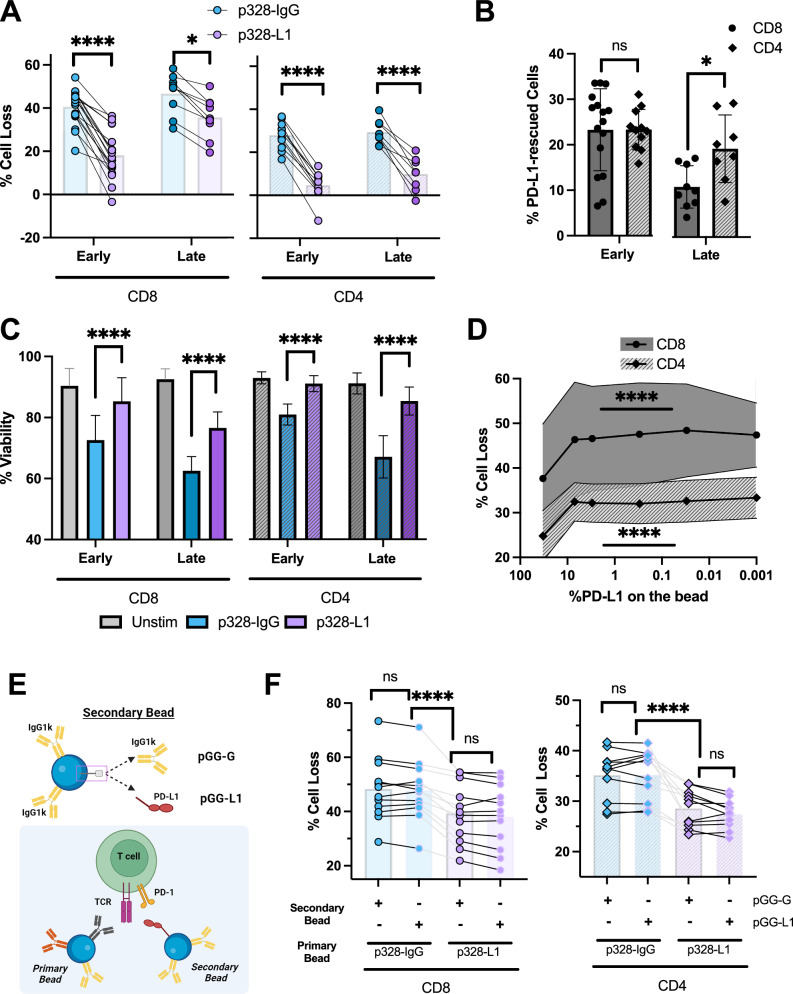
PD-L1 induces dose-dependent RICD rescue in CD4+ and CD8 + T cells by functioning in the “immunological synapse”. **A** CD8+ or CD4 + T cells were restimulated on Day 4 or Day 11 with p328-IgG of p328-L1 beads at a 1:1 bead:cell ratio and monitored for cell loss via flow cytometry 24 hr later using propidium iodide. *N* = 8 CD4+ early, *N* = 11 CD4+ late, *N* = 9 CD8+ early, *N* = 18 CD8+ late. **B** The difference in % cell loss between p328-IgG and p328-L1 beads in (**A**) is plotted for each donor, cell type, and cell stage evaluated. (same N as (**A**). **C** RICD assays set-up in (**A**) were examined on the flow cytometer using propidium iodide to calculate the percentage of live cells remaining after 24 hr of restimulation. *N* = 7 CD4+ early, *N* = 9 CD4+ late, *N* = 12 CD8+ early, *N* = 8 CD8+ late. **D** PD-L1 dose response was determined using the standard RICD setup amd a new bead set that gradually decreased PD-L1 concentration on the bead from 33% to 0%. Day 4 CD4+ and CD8 + T cells were utilized in this experiment. *N* = 12 CD4 + , *N* = 10 CD8 + , statistical analysis was calculated with two-way ANOVA. **E** Diagram representing RICD setup and creation of secondary bead set that was ligated 100% to IgG1κ (control secondary bead) or 67% IgG1κ and 33% recombinant PD-L1, matching the PD-L1 protein concentration on the p328-L1 primary bead. **F** Day 4 CD8+ and CD4 + T cells were restimulated with a 2:1 bead:cell ratio comprising a 1:1 ratio of the primary bead and 1:1 ratio of the secondary bead. RICD was monitored as in (**A**) *N* = 12.

Given high expression of PD-1 by day 2 after T cell activation, we next investigated the effect of PD-1 engagement on RICD sensitivity in early effectors restimulated with our pAPC. Using day 4 (early) CD8+ and CD4 + T cells, p328-L1 beads reliably and significantly decreased cell loss induced by restimulation compared to IgG control beads (Fig. 2A). Although CD4 + T cells showed reduced levels of RICD as a whole when compared to CD8 + T cells, both cell types experienced ~20% rescue with p328-L1 bead restimulation relative to p328-IgG beads (Fig. 2B). By contrast, PD-1 expression was relatively low in late-stage, terminally differentiated effector T cells (day 11) (Fig. 1A). Interestingly, late stage CD8 + T cells showed a dampened RICD rescue effect with p328-L1 beads relative to CD4 + T cells and early-stage T cells (Fig. 2A, B). Comparatively heightened levels of PD-1 expression in CD4 + T cells, or differences in CD4+ vs CD8 + PD-1 signaling pathways, may partially account for this sustained protective effect. These data suggest that PD-L1 engagement can induce a meaningful reduction in RICD sensitivity even when PD-1 expression is relatively low. Although the “% cell loss” calculation is thought to provide a more accurate assessment of cell death by quantifying the absolute number of live cells remaining after restimulation, we also observed a corroborating increase in cell viability when restimulating with p328-L1 vs. p328-IgG beads in all cell types and conditions assessed (Fig. 2C). To determine whether PD-1-dependent RICD protection was titratable, we performed a dose curve by varying concentrations of PD-L1 on pAPC beads. Cell loss increased in both CD4+ and CD8 + T cells as the PD-L1 concentration on beads decreased, with the most significant jump observed from 100% to 25% of the original PD-L1 bead content (Fig. 2D). We next asked whether PD-1 engagement must occur in close proximity to the TCR and CD28 to enforce RICD resistance, simulating signaling within the immunological synapse (IS). In addition to p328-IgG and p328-L1 beads used for primary restimulation, we introduced a secondary bead decorated only with IgG1κ or PD-L1-Fc at an equivalent concentration (Fig. 2E). In contrast to p328-L1 beads alone, concomitant restimulation using the primary p328-IgG bead with p328-L1 on the secondary bead did not reduce RICD (Fig. 2F), suggesting PD-1 must be engaged near the TCR and CD28 in the IS to effectively reduce RICD. In both CD4+ and CD8 + T cells, the secondary PD-L1 bead provided no additional effect on RICD, regardless of the primary bead used. Taken together, these data indicate that PD-1 engagement can protect clonally expanding (and to a lesser extent terminally differentiated) T cells from RICD. This effect is both dose-dependent and reliant on PD-1 co-localization near the TCR and CD28, consistent with prior findings [42].

### PD-1 signaling decreases apoptosis after T cell restimulation

**Fig. 3:**
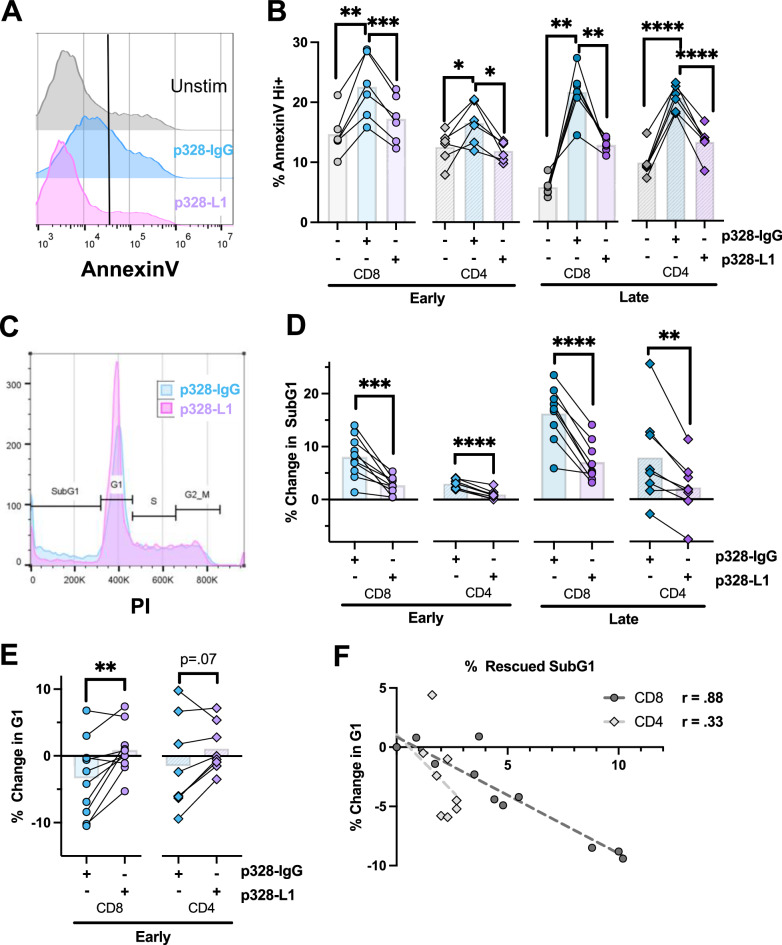
Restimulated CD4+ and CD8 + T cells experience less apoptosis when PD-1:PD-L1 is engaged. **A** Representative histogram of data displaying demarcation of AnnexinV^hi^ cells -/+ bead restimulation. **B** Day 4 and Day 11 CD4+ and CD8 + T cells were left unstimulated or were restimulated with p328-IgG or p328-L1 beads for 6 hr before being stained with AnnexinV and propidium iodide and read on the flow cytometer. *N* = 6. **C** Representative flow cytometric histogram of PI cell cycle phases based on DNA content. **D** Day 4 and Day 11 CD4+ and CD8 + T cells were left unstimulated or were restimulated with p328-IgG or p328-L1 beads for 18 hr before being permeabilized and stained with propidium iodide to determine % of cells in the subG1 phase. *N* = 8 CD4+ early, *N* = 8 CD4+ late, *N* = 10 CD8+ early, *N* = 9 CD8+ late. **E** Analysis using gated cells from (**D**) was performed to quantify changes in the G1 phase. **F** Cell phase characterization from (**D**) was analyzed to determine the PD-L1 induced difference in subG1 and G1 phases.

Cell loss assays used to determine assess RICD sensitivity are based on discrimination of live vs. dead cells with propidium iodide (PI) staining based on plasma membrane integrity, which is not a strict readout of apoptosis. To examine whether PD-1 engagement during restimulation specifically reduces apoptosis, we utilized flow cytometry to assess phosphatidylserine exposure via Annexin V staining and DNA fragmentation via subG1 PI cell cycle analysis as markers of early and late-stage apoptosis, respectively. Both CD4+ and CD8+ early-stage T cells demonstrated a significant increase in AnnexinV^hi^ cells six hours after p328-IgG bead restimulation (Fig. 3A, B). Conversely, p328-L1 bead restimulation in the same donor showed a significant drop in AnnexinV^hi^ cells that was similar to unstimulated T cells, confirming that PD-1 signaling specifically prevented apoptosis in clonally expanding cells. Cell cycle analysis also demonstrated a significant reduction in apoptotic subG1 cells (i.e. cells with fragmented DNA) with p328-L1 bead stimulation relative to p328-IgG beads (Fig. 3C, D). Similar results were observed in terminally-differentiated “late” T cells (Fig. 3B, D). Regardless of when T cells were restimulated, CD4 + T cells experienced reduced apoptotic sensitivity relative to CD8 + T cells, congruent with our initial RICD data (Fig. 2A).

A previous study determined that terminally differentiated T cells cycling through S phase are particularly sensitive to RICD [43]—however, clonally expanding T cells have never been evaluated in this manner. As expected, the percentage of cells in S phase was higher in clonally expanding T cells relative to late-stage effectors, reflecting increased proliferation (Supplementary Fig. 3). We then analyzed whether the degree of rescue induced by p328-L1 beads correlates with the percentage of cells lost from S phase. Regardless of bead stimulation, we found no definitive correlations in CD4+ or CD8+ cells; in fact, a minor increase in S phase cells was observed with p38-IgG1 vs. p38-L1 bead stimulation (Supplementary Fig. 3). However, we did note a positive inverse correlation between the percentage of apoptotic subG1 cells and decreased S phase cells when stimulated with soluble anti-CD3 (OKT3) alone (Supplementary Fig. 4), suggesting CD28 costimulation may influence cell cycle progression differently than TCR engagement alone. Interestingly, we did note a significant increase in cells exiting G1 phase with p328-IgG versus p328-L1 bead restimulation in CD8 + T cells, with a modest trend noted in CD4+ cells as well (Fig. 3E). We also found a positive correlation in both subsets between the proportion of cells spared from RICD by PD-L1 and the percentage of cells that remain in G1 (Fig. 3F), implying that PD-1 may help protect early activated T cells from premature apoptosis in part by limiting CD28-induced cell cycle entry.

### Enhanced RICD with CD28 costimulation is reversed by PD-1 co-ligation

**Fig. 4:**
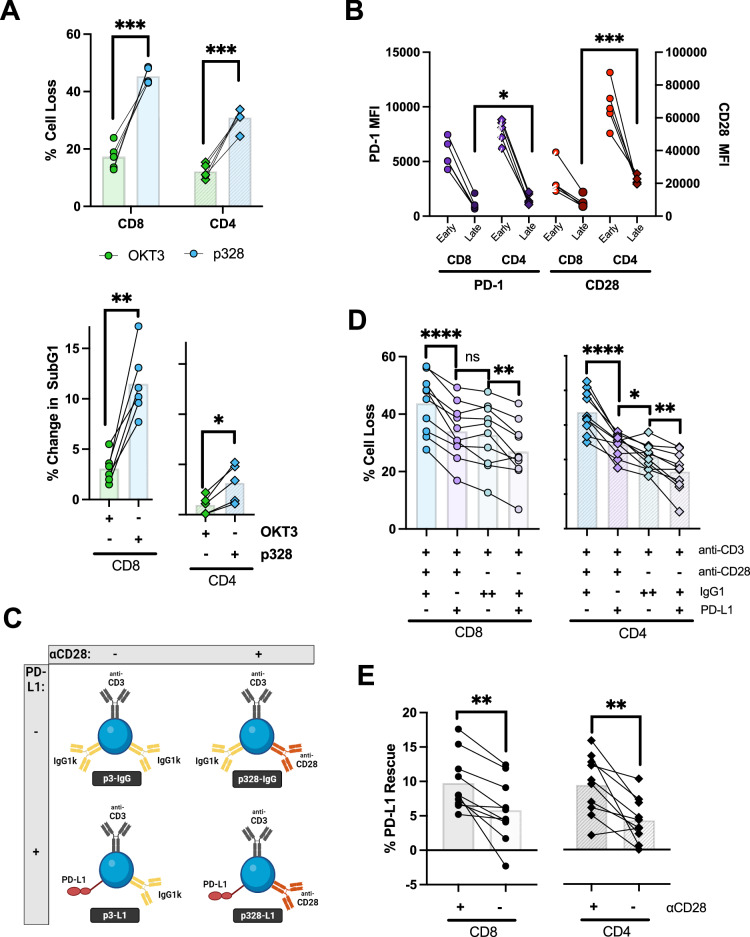
PD-L1 abolishes the increased RICD sensitivity incurred by CD28 signaling during TCR restimulation. **A** CD8+ or CD4 + T cells were restimulated on Day 4 with soluble anti-CD3 (OKT3) at 100 ng/mL or anti-CD3/CD28 coated beads at a 1:1 bead:cell ratio. Cell loss/apoptosis was quantified 18–24 hr later after propidium iodide staining and flow cytometry. N = 5 CD4+ early, N = 6 CD4+ late, *N* = 5 CD8+ early, *N* = 6 CD8+ late. **B** Day 4 and Day 11 CD4+ and CD8 + T cells from *N* = 5 individual donors were stained for CD28 and PD-1 expression and compared via flow cytometry. **C** Diagram illustrating restimulation beads conjugated with or without anti-CD28, with p328-IgG used to maintain total protein concentration. **D** CD8+ or CD4 + T cells were restimulated on Day 4 with beads illustrated in (**C**) at a 1:1 bead:cell ratio, monitored for cell loss 24 hr later using propidium iodide using flow cytometry. **E** The difference in cell loss induced by restimulation with beads -/+ anti-CD28 (leaving anti-CD3 + /- PD-L1-Fc constant) was plotted for every donor (*N* = 10).

While CD28 signaling is instrumental in T cell activation, its role in RICD remains nebulous. Mature T cells do not require co-stimulation for RICD induction, and CD28 co-ligation has been shown to augment or prevent TCR-induced apoptosis in conflicting reports [38, 44]. TCR and CD28 re-engagement is more relevant in the context of clonal expansion, and CD28 has been shown to be a critical target for PD-1 signal regulation [45]. Relative to OKT3 restimulation alone, anti-CD3/CD28 Ab-coated beads markedly increased cell loss in day 4 CD8+ and CD4 + T cells by inducing apoptosis, as monitored by PI exclusion and cell cycle analyses (Fig. 4A, B). In our in vitro T cell cultures, both PD-1 and CD28 are highly expressed in early-stage T cells, whereas expression of both receptors drops markedly in late-stage effectors (Fig. 4B). Notably, CD28 and PD-1 expression are ~1.5–3x higher in terminally differentiated CD4 + T cells compared to CD8+ cells, which might help to explain the enhanced RICD rescue effect of p328-L1 beads seen in late-stage CD4 + T cells (Fig. 2A). To specifically assess how CD28 signaling influences RICD sensitivity in early expanding effector T cells, we created additional pAPC beads that did not include anti-CD28 for use in RICD assays (dubbed p3-IgG and p3-L1) (Fig. 4C). We found that PD-1 engagement with beads containing PD-L1-Fc decreases RICD with and without concurrent CD28 signaling (Fig. 4D). However, the proportion of cells rescued from RICD in the presence of anti-CD28 co-stimulation was significantly greater than those stimulated with anti-CD3 alone (Fig. 4E). Intriguingly, PD-L1 completely abolished the additional cell loss incurred from CD28 co-engagement in CD8 + T cells, with a less pronounced but similar effect in CD4 + T cells. These data imply that CD28 engagement during TCR restimulation boosts RICD sensitivity in early activated T cells, which is effectively counteracted by concomitant PD-1 ligation.

### PD-1 modulates TCR/CD28 signal strength during restimulation of early expanding T cells

**Fig. 5:**
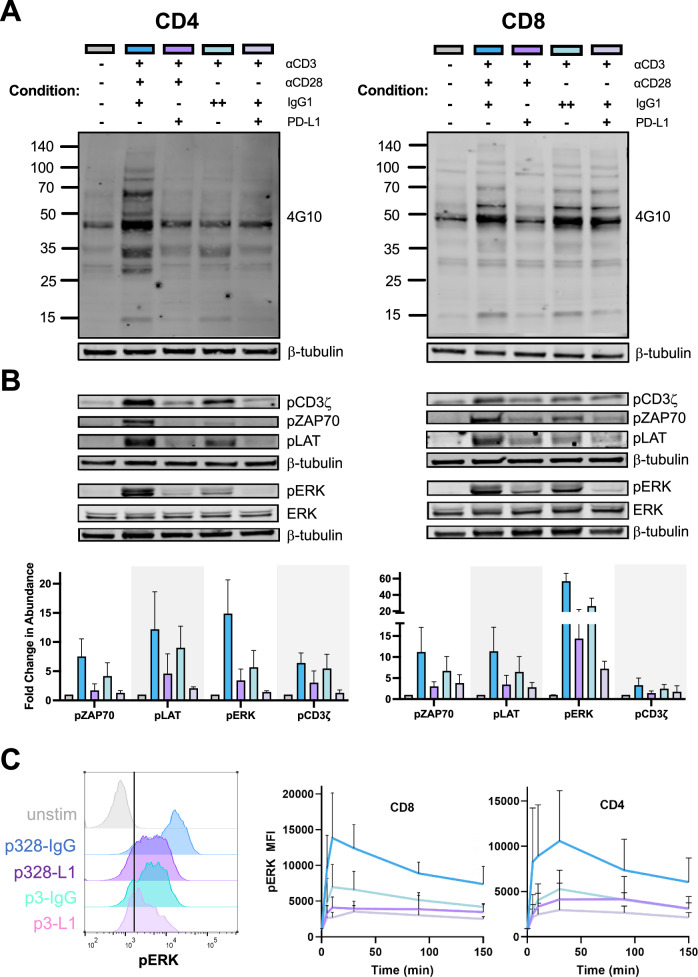
PD-1 ligation modulates signal transduction downstream of TCR/CD28 restimulation in early effector T cells. **A**, **B** Day 4 CD4+ or CD8 + T cells deprived of IL-2 for 24 hr were starved in PBS + 2% BSA for 2 hr prior to bead restimulation. Whole cell lysates made after 5 min of restimulation were separated by SDS-PAGE and immunoblotted with 4G10 anti-phosphotyrosine Ab (**A**) or individual Abs directed against proximal signaling proteins (**B**). Blots are representative of repeat experiments in 3 donors. Relative phosphoprotein signals were quantified by spot densitometry, normalized to β-tubulin as a loading control. **C** Representative histogram (left) for flow cytometric quantification of phospho-ERK levels -/+ bead restimulation in CD4 + T cells. Graph depicts pERK levels over time in CD4+ and CD8 + T cells prepared as in (**A**). N = 4.

Because the PD-1-mediated rescue effect on RICD in early-stage T cells appeared to involve modulation of both CD3 and CD28 signaling, we next explored the effect of PD-1 engagement on intracellular signaling events downstream of TCR restimulation -/+ CD28 ligation by immunoblotting. Using the 4G10 antibody to detect changes in global protein tyrosine phosphorylation, we observed marked decreases in phosphorylation of multiple proteins when PD-L1-Fc was present on the restimulation bead in both CD8+ and CD4 + T cells, relative to beads lacking PD-L1 (Fig. 5A). These differences were more pronounced with simultaneous CD28 signaling, congruent with relative RICD levels noted in Fig. 4D. To interrogate the effect of PD-L1 on distinct proximal signaling components, we probed the same set of lysates with phospho-specific antibodies. As expected, PD-L1 ligation during restimulation dramatically dampened the phosphorylation of CD3ζ, ZAP70, and LAT, as well as ERK (Fig. 5B). Although PD-L1 dampened proximal TCR signaling induced by anti-CD3 alone, the effect was clearly more pronounced with CD28 co-engagement, which substantially increased the phosphorylation of all proteins examined. Given the reliable effect of PD-1 engagement in blunting ERK phosphorylation, and its possible implications for cell cycle progression [46], we analyzed phospho-ERK levels over a timecourse post-restimulation using intracellular flow cytometry. Post-TCR restimulation alone, ERK phosphorylation peaked at 10 min in CD8 + T cells and 30 min in CD4+ cells, dwindling to low levels by 2.5 hr (Fig. 5C). By contrast, restimulation with both anti-CD3 and anti-CD28 produced a dramatic, sustained increase in pERK over the course of the experiment, continuing at least 5 hr post-restimulation (data not shown). The addition of PD-L1 markedly reduced ERK phosphorylation to comparable levels regardless of CD28 co-ligation. Altogether, these data indicate that robust TCR signal transduction enhanced by concomitant CD28 engagement is effectively dampened by PD-L1 co-ligation during restimulation of clonally expanding T cells, suggesting PD-1 reduces RICD sensitivity during this period by modulating both TCR and CD28 signal strength.

### PD-1 engagement alters the expression of pro- and anti-apoptotic molecules affected by TCR/CD28 restimulation

**Fig. 6:**
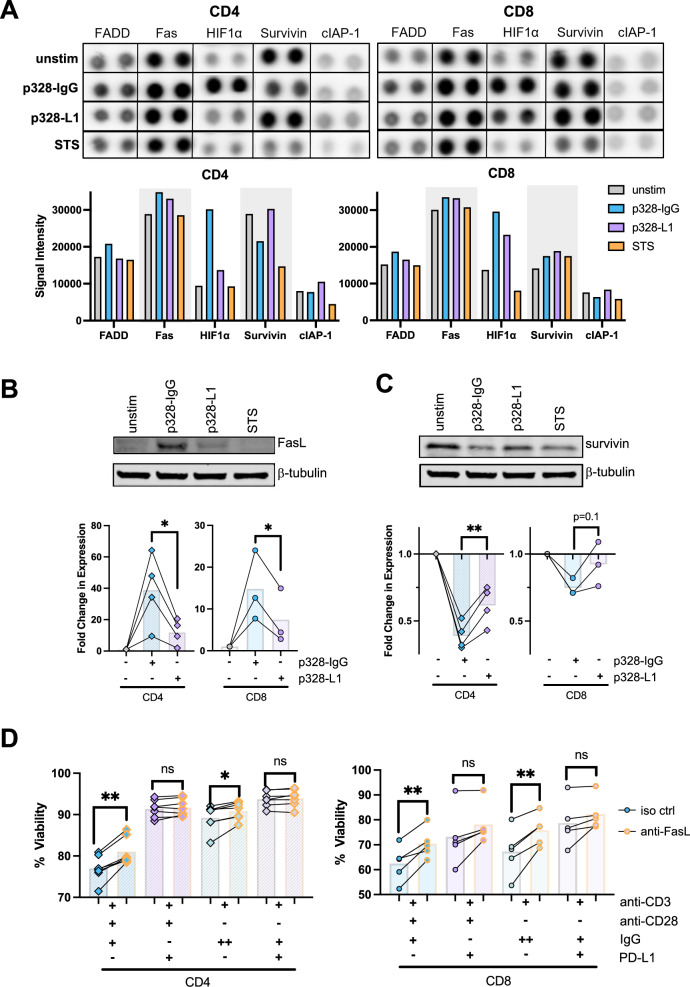
PD-1 signaling alters pro- and anti-apoptotic molecules during to restimulation to enhance survival. **A** Day 4 CD4+ and CD8 + T cells (*N* = 4 donors each) were restimulated with p328-IgG or p328-L1 beads at a 1:1 bead:cell ratio for 5 hr prior to lysate collection. Control cells were left unstimulated or treated with 1 μM STX. After cell lysis and protein standardization, donor lysates were pooled for each stimulation condition and analyzed on the apoptosis protein array. Representative spots for differentially expressed proteins are shown. Protein signal intensity from membrane imaging was analyzed and normalized using QuickSpots software. T cells were restimulated as in (**A**), lysed in RIPA buffer and separated by SDS-PAGE to quantify FASL (**B**) and survivin (**C**). Data are representative of experiments for 3–4 separate donors. (**D**) Day 4 CD4+ and CD8 + T cells were treated with 10 μg/mL anti-FASL blocking antibody for 1 hr prior to restimulation with the quad bead set. Cells were restimulated for 24 hr and stained with propidium iodide; % viability was assessed by flow cytometry. *N* = 7 CD4 + , *N* = 5 CD8 + .

Given the substantial effect of PD-1 in tempering the TCR/CD28 signaling cascade during restimulation, we next interrogated downstream effects on the expression of molecules implicated in apoptosis regulation. We employed an apoptosis-proteome sandwich array to measure changes in the expression of 35 pro- and anti-apoptotic proteins in pooled day 4 CD8 + T cells versus CD4 + T cells (*n* = 4 donors each) restimulated for 5 hr with beads -/+ PD-L1-Fc, relative to cells that were not restimulated or treated with staurosporine (STS) as a positive control for apoptosis induction (Supplemental Fig. 5). Although the expression of many proteins remained similar, several proteins demonstrated significant differential expression changes between p328-IgG versus p328-L1 restimulation (Fig. 6A). For example, an increase in hypoxia-inducible factor 1α (HIF-1α), a TCR-induced protein involved in T cell differentiation and metabolism [47], was blunted by concomitant PD-L1 ligation during restimulation, particularly in CD4 + T cells. Conversely, high expression of the anti-apoptotic protein survivin (encoded by *BIRC5*) in unstimulated CD4 + T cells was maintained with p328-L1 bead restimulation, but substantially decreased with p328-IgG bead restimulation and STS treatment. Minor increases were also measured in the pro-survival protein cellular inhibitor of apoptosis protein 1 (cIAP-1) with PD-L1 restimulation in CD8+ and CD4 + T cells, whereas FADD and FAS expression were modestly increased with p328-IgG bead restimulation only (Fig. 6A).

Given the importance of FAS signaling in RICD of late-stage effector T cells, we also investigated FASL induction after bead restimulation. As expected, immunoblotting showed that FASL expression was significantly upregulated upon TCR restimulation using beads -/+ anti-CD28 (Fig. 6B). Strikingly, PD-L1-ligated beads largely blunted FASL induction in both CD4+ and CD8 + T cells. PD-L1-mediated preservation of survivin expression was also confirmed and quantified by immunoblotting (Fig. 6C), confirming our proteome array results.

FASL:FAS ligation is a classic apoptosis-inducing pathway in terminally-differentiated T cells [30], but its relevance in early-stage T cells is incompletely understood. We found that pre-incubation with an anti-FASL blocking antibody significantly increased cell viability after restimulation in both CD4+ and CD8 + T cells when using anti-CD3 coated beads -/+ anti-CD28 (Fig. 6C). In contrast, FASL blockade had no significant impact on RICD induced by PD-L1-conjugated beads, likely due to reduced FASL induction with PD-1:PD-L1 signaling. The relatively modest protective effect observed with FASL blockade post-restimulation might be explained in part by a stimulatory role for FAS-FASL signaling during clonal expansion, before FAS fully converts to a pure death receptor [48, 49]. Nevertheless, these data suggest that PD-1 promotes RICD resistance in part by blunting TCR-induced FASL upregulation and survivin downregulation, providing mechanistic insight into how PD-1 enables optimal clonal expansion by tuning TCR/CD28 signaling to preclude premature apoptosis.

## Discussion

Regulatory programs that govern T cell population dynamics are critical in determining the magnitude and potency of the effector T cell response to foreign antigen. RICD helps curb T cell expansion to prevent lymphoproliferation and associated tissue damage, but the complexities of RICD sensitivity regulation, especially during clonal expansion, require further investigation [1, 2]. A vital prerequisite for RICD is robust TCR engagement and proximal signaling. Given our recent findings on TIM-3, a co-inhibitory receptor that differentially regulates RICD sensitivity over time [28], we hypothesized that other co-inhibitory molecules like PD-1 could protect T cells from premature RICD during clonal expansion by dampening TCR signal strength post-restimulation. The discovery and characterization of PD-1 in the early 1990s represented a seminal step forward in understanding how co-inhibitory receptors impact T cell function, helping to catalyze an explosion of checkpoint inhibition strategies for cancer immunotherapy [50]. While originally presumed to be a programmed death-inducing protein, myriad studies have found that PD-1 does not signal apoptosis of thymocytes or mature T cells [51, 52], and may in fact promote T cell survival in response to chronic antigen exposure or bystander activation [53, 54]. Indeed, our work definitively demonstrates that normal upregulation of PD-1 early after initial T cell activation can promote cell survival during clonal expansion by enforcing RICD resistance.

Proxy APCs (pAPC) constructed from magnetic beads coated with agonistic antibodies or recombinant proteins offer a common method employed for T cell activation and re-stimulation in the derivation of adoptive T cell therapies [55]. Given our early data suggesting a role for PD-1 in T cell survival when restimulating T cells in the presence of PD-L1-expressing irradiated autologous PBMC (Fig. 1B), we formulated pAPCs decorated -/+ PD-L1-Fc to simultaneously trigger PD-1 signaling with TCR/CD28 restimulation. Although a prior report showed that PD-L1-Fc or CTLA-4-Ig can actually outcompete anti-CD3 when added simultaneously for bead coupling [56], we observed similar levels of anti-CD3 per bead -/+ PD-L1-Fc (data not shown), perhaps because we always included an equivalent amount of Fc-matched isotype control antibody during the coupling procedure. Our results revealed that PD-1 signaling reduces RICD for both CD4+ and CD8 + T cells even when PD-1 expression is relatively low, provided that relatively high concentrations of PD-L1 were delivered in proximity to the artificial immunological synapse (IS) formed between bead and cell. The proximity of PD-1 is tightly regulated in the IS, with signal inhibition only occurring when PD-L1 is presented at the appropriate distance from anti-CD3 and anti-CD28 at sufficient concentrations [42, 57]. Indeed, the PD-1-mediated protective effect on RICD was lost with lower PD-L1 concentrations on pAPC, or with simultaneous delivery of PD-L1 on a separate bead. The latter finding correlates with previous literature demonstrating the need for ligand binding to PD-1 to induce clustering and proper localization to the IS [58]. Interestingly, this study also showed that elevated PD-1 concentration in the IS is preferentially determined by PD-L2, which binds with higher affinity versus PD-L1 [59]. While PD-L2 was not tested here, one could envision more potent RICD resistance facilitated by PD-L2 even at decreased concentrations on pAPC, with additional consequences for subsequent effector functions [60].

Early work suggested that PD-1:PD-L1 interactions can drive increased apoptosis of alloreactive, viral or tumor-specific T cells to negatively regulate their survival [36, 61–63]. However, subsequent work clearly showed that while PD-1 signaling can induce cell cycle arrest in T cells, it plays no direct role in apoptosis induction [46, 64]. We now appreciate that PD-1 serves a more nuanced role in tuning effector responses by preventing immunopathology, suppressing autoimmunity, and limiting hyperactivation with chronic antigen stimulation, which may actually help distinct populations of exhausted T cells (Tex) survive RICD [65]. Using AnnexinV staining and cell cycle analysis, we directly demonstrated that PD-1 engagement reduced apoptosis following TCR/CD28 restimulation. Moreover, we uncovered a correlation between PD-L1-afforded RICD resistance and an increase in G1 phase cells. An earlier report found PD-1 signaling could slow progression through the G1 phase via inhibition of the PI-3K-AKT and RAS-MEK-ERK pathways and reduced expression of S-phase kinase-associated protein-2 (Skp2), a critical ubiquitin ligase that upregulates cell cycle kinases [46]. Additionally, PD-1 inhibited phosphorylation of Smad3 and Rb, both of which impair transcription factors required for cell cycle progression. Interestingly, IL-2 can partially restore expression of Skp2 and T cell proliferation by reversing the specific PD-1-mediated inhibitory effect on MEK-ERK (but not PI-3K-AKT) signaling. Notably, these differences were observed during initial priming of T cells, when enforced PD-1 signaling drastically limits T cell activation, IL-2 production, and growth [41] (Fig. 1). Once clonal expansion is underway, recently activated T cells may utilize PD-1 and IL-2 signaling to strike a balance between effective cell cycle progression and proliferation, without premature apoptosis due to antigen restimulation.

Cell cycle analysis also revealed a prominent role for CD28 signaling in potentiating RICD in early stage effector T cells with TCR re-engagement. Previously, the influence of CD28 on RICD sensitivity was nebulous, and often considered irrelevant for terminally-differentiated effector T cells [38, 44, 66]. However, recently activated T cells upregulate expression of CD28 and other co-signaling molecules, during the time period when repeated encounters with APCs are possible in secondary lymphoid tissues [67]. Restimulation in this context likely relies on a balance of co-stimulatory and co-inhibitory signals to properly tune TCR signaling and limit RICD. Indeed, we found that PD-1 dampened CD28-dependent amplification of phosphorylation of several proximal TCR signaling molecules (Fig. 5), revealing why early effectors experience significantly more PD-L1-mediated rescue from RICD with concomitant CD28 signaling during TCR restimulation (Fig. 4). Although PD-1-dependent recruitment of SHP-2 phosphatase has been shown to dampen TCR/CD28 signaling [64], we noted that SHP-2 phosphorylation was similar in restimulated T cells -/+ PD-L1-Fc, and that SHP-2 inhibition surprisingly had no significant effect on RICD sensitivity regardless of stimulus (data not shown). More work is required to elucidate how PD-1 ligation ultimately modulates TCR/CD28 signaling during RICD, which may involve additional mediators including PAG, VRK-2, and SAP [68–70]. Indeed, SAP expression is essential for RICD sensitivity, and SAP was previously shown to block PD-1 function [71].

We posit that relative RICD resistance in clonally expanding T cells is enforced in part by co-inhibitory receptors that enable optimal proliferation without detrimental hyperstimulation. Both TIM-3 and CTLA-4 are upregulated after initial T cell stimulation and have previously been linked to RICD resistance [28–30], the latter being definitively connected to CD28 antagonism. As such, our findings herein add to a growing body of literature attempting to deconvolute whether PD-1 preferentially targets TCR or CD28-specific downstream signaling cascades [45, 72]. There is noteworthy clinical significance in this area, as reports demonstrate PD-1 checkpoint blockade actually requires simultaneous CD28 signaling [73]. Interestingly, progenitor exhausted T cells (Tpex) responsible for PD-1 blockade efficacy require PD-1 expression, and can initiate self-renewal or production of terminal exhausted T cells (Tex) based on the strength of CD28 costimulation [74]. Our findings imply that PD-1 may protect this critical Tpex population from RICD, enabling their persistence in vivo.

We also found that PD-1 ligation during TCR/CD28 restimulation changes the expression of several anti- and pro-apoptotic molecules. For example, PD-1 signaling suppressed the induction of HIF-1α during restimulation, particularly in CD4 + T cells. Intriguingly, a recent report found that ablation of HIF1α in both human and mouse CD4 + T cells resulted in attenuated RICD, which was potentially linked to reduced glycolysis and IFN-γ secretion [75]. Prior work from our group and others established that both IFNγ and glycolysis promote RICD in human T cells [22, 26], and PD-1 ligation is known to inhibit glycolysis in favor of fatty acid oxidation [76]. By contrast, PD-L1 ligation prevented downregulation of the anti-apoptotic protein survivin observed in both CD4+ and CD8 + T cells restimulated with p328-IgG beads. Survivin is a member of the Inhibitor of Apoptosis (IAP) family best known for its elevated expression in cancer cells, representing an attractive but challenging therapeutic target for tumor eradication [77, 78]. Survivin also plays an important role in T cell homeostasis and survival. Survivin is upregulated through the first six days after initial T cell activation (more so in memory versus naïve T cells), aiding in S-phase transition and apoptosis resistance via OX40- and PI-3K-induced BCL2 expression [79, 80]. Indeed, disrupting survivin through pharmacological inhibition or CRISPR/Cas9-mediated deletion severely restricted growth and viability in our T cell cultures (data not shown). We conclude that PD-1 signaling likely enforces RICD resistance in part by sustaining high survivin expression in clonally expanding T cells, but more studies are required to fully decipher this mechanism.

FAS-induced apoptosis has long been connected to RICD in mature effector T cells, particularly in terminally-differentiated CD4 + T cells. However, more recent work revealed an early non-apoptotic role for FAS as a costimulatory molecule [81, 82]. In fact, FASL expression on memory T cells can accelerate differentiation of naïve T cells after initial antigen encounter, enhancing their proliferation [83]. We detected a marked decrease in FASL induction with p328-L1 bead restimulation relative to p328-IgG beads. As a result, FASL blockade only enhanced the viability of cells restimulated with p328-IgG beads—no significant changes were noted with PD-L1-loaded beads, likely due to poor FASL upregulation (Fig. 6). Interestingly, cell loss by flow cytometric quantification of live cells over a constant time period showed no appreciable difference (data not shown). While the percent cell loss calculation is thought to be the most sensitive readout for RICD, the number of viable cells remaining after TCR restimulation ultimately represents a net balance between cell proliferation and death. As FAS transitions from costimulatory to pro-apoptotic function during clonal expansion, FASL blockade on day 4 post-activation may paradoxically decrease both cell proliferation and apoptosis in distinct effector subsets, resulting in only a modest increase in viability. Indeed, T cells derived from naïve versus memory cell subsets are differentially sensitive to FAS-induced apoptosis [84]. Future work is required to dissect the multi-faceted role of FAS:FASL signaling in early-stage effector T cells, and how PD-1 signaling affects its non-apoptotic and apoptotic functions.

As our mechanistic understanding of PD-1 directed immunotherapies advances, so too must our knowledge of normal PD-1 biology to improve checkpoint blockade efficacy and reduce irAEs. Here we unveil a novel protective role for PD-1 in modulating RICD sensitivity during clonal expansion of both CD4+ and CD8+ human T cells. Our results clearly indicate PD-1 can repress both TCR and CD28 signaling to promote RICD resistance. In the context of PD-1/PD-L1 blockade, we must consider this homeostatic role in preserving optimal nascent T cell responses. Future research should investigate survival dynamics of PD-1 + T cells before and after checkpoint inhibition, and how this may effect immunotherapy efficacy and bystander T cell dysregulation. Although PD-1 blockade is best known for rejuvenating CD8 + T cells in cancer immunotherapy, CD4 + T cells are also impacted. PD-1 signaling can suppress CD4 + T cell effector function and induce regulatory T cell phenotypes [85, 86], and PD-1 blockade can enhance the immune response to class II MHC-bearing Hodgkin lymphoma tumors in a manner dependent upon cytotoxic CD4 + T cells [87]. Additionally, as adoptive T cell therapies (ACTs) using TILs or CAR-T cells become standardized cancer treatments, it will be imperative to optimize activation and restimulation culture conditions to expand billions of effector T cells required for infusion into patients. Monitoring and minimizing RICD in this context is crucial, which may require balancing the delivery of TCR and costimulatory/coinhibitory stimuli to preserve long-lived T cells capable of robust effector function and multipotency/self-renewal.

## Supplementary information

Supplemental Figures 1-5, Supplemental Tables 1-3

Supplemental Material - Blots

## Data Availability

Datasets not included in this article are available from the corresponding author on request.
