# Supplementary material for: PD-1 protects expanding human T cells from premature restimulation-induced cell death by modulating TCR and CD28 signaling
Source: Cell Death Dis. 2026 Feb 26;17(1):272. doi: 10.1038/s41419-026-08530-6 (PMC13004874; doi:10.1038/s41419-026-08530-6)
Supplement: Supplementary file 1 — Supplemental Figures 1-5, Supplemental Tables 1-3 [file 41419_2026_8530_MOESM1_ESM.docx]

**Supplemental Material**

**PD-1 protects expanding human T cells from premature restimulation-induced cell death by modulating TCR and CD28 signaling.**

Katherine P. Lee^1^, Sara Elster^1^, Benjamin Epstein^1^, Camille M. Lake^1^, Andrew L. Snow^1*^

^1^Department of Pharmacology & Molecular Therapeutics, Uniformed Services University of the Health Sciences, Bethesda, MD, USA

Supplemental Figure 1. Gating strategy and % cell loss determination for RICD assays. Data represent day 10 effector T cells -/+ OKT3 restimulation for 24 hours. Cell loss was measured according to the indicated formula, using propidium iodide staining to distinguish live/dead cells.


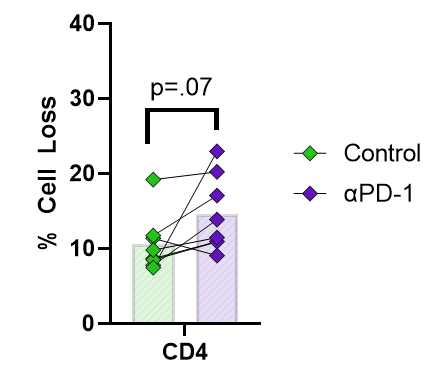

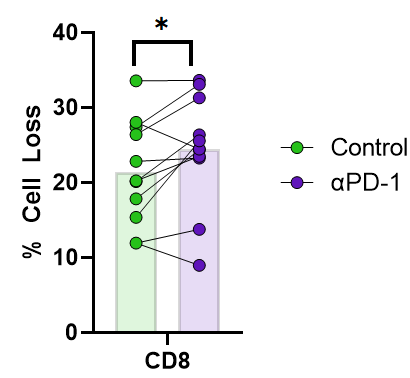


Supplemental Figure 2. Blockede of PD-1 on purified clonally expanding T cell cultures has little effect on RICD. RICD assay was set-up with Day 4 T cells that were incubated with anti-PD-1 or IgG1κ at 10 μg/mL for one hour prior to restimulation with OKT3 at 100 ng/mL. Cell loss was recorded with propidium iodide staining on the flow cytometer.


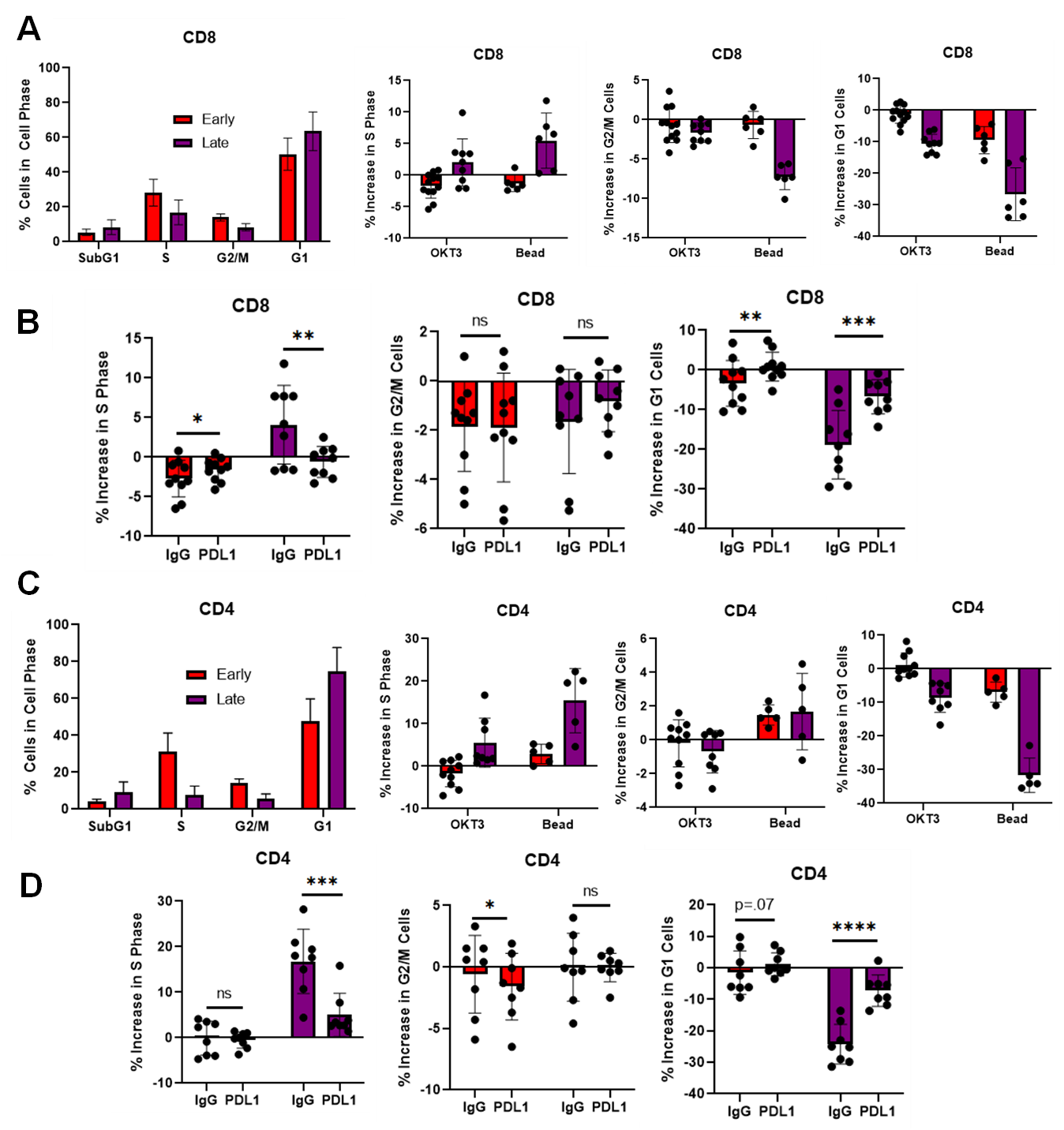
Supplemental Figure 3. Cell cycle analysis overview of early and late-stage CD4+ and CD8+ T cells and the effects of pCD328-L1 beads on S, G2/M, and G1 phases.

PI cell cycle analysis was performed to assess additional correlations regarding the overall state of cell cycle progression in early (red bars) and late (purple bars) stage CD8+ **(A-B)** and CD4+ **(C-D)** T cells following restimulation with OKT3 vs. p328-IgG beads (A, C), or p328-IgG vs. p328-L1 beads (B, D).


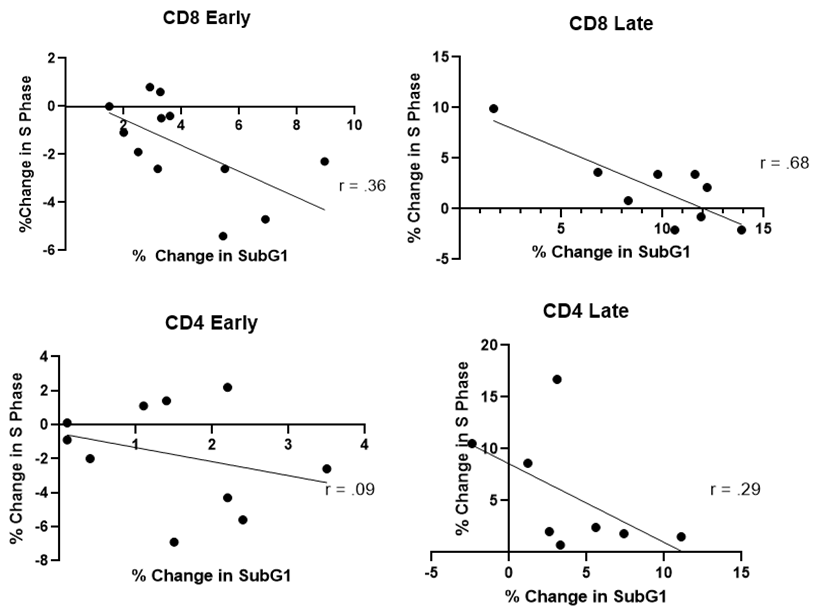
Supplemental Figure 4. Correlation curves comparing S phase and subG1 in OKT3-restimulated T cells. Cells were set-up and restimulated as in Figure 3. Correlations highlight the decrease in S phase and corresponding increase in subG1 phase experienced upon OKT3-restimulation at 100 ng/mL.


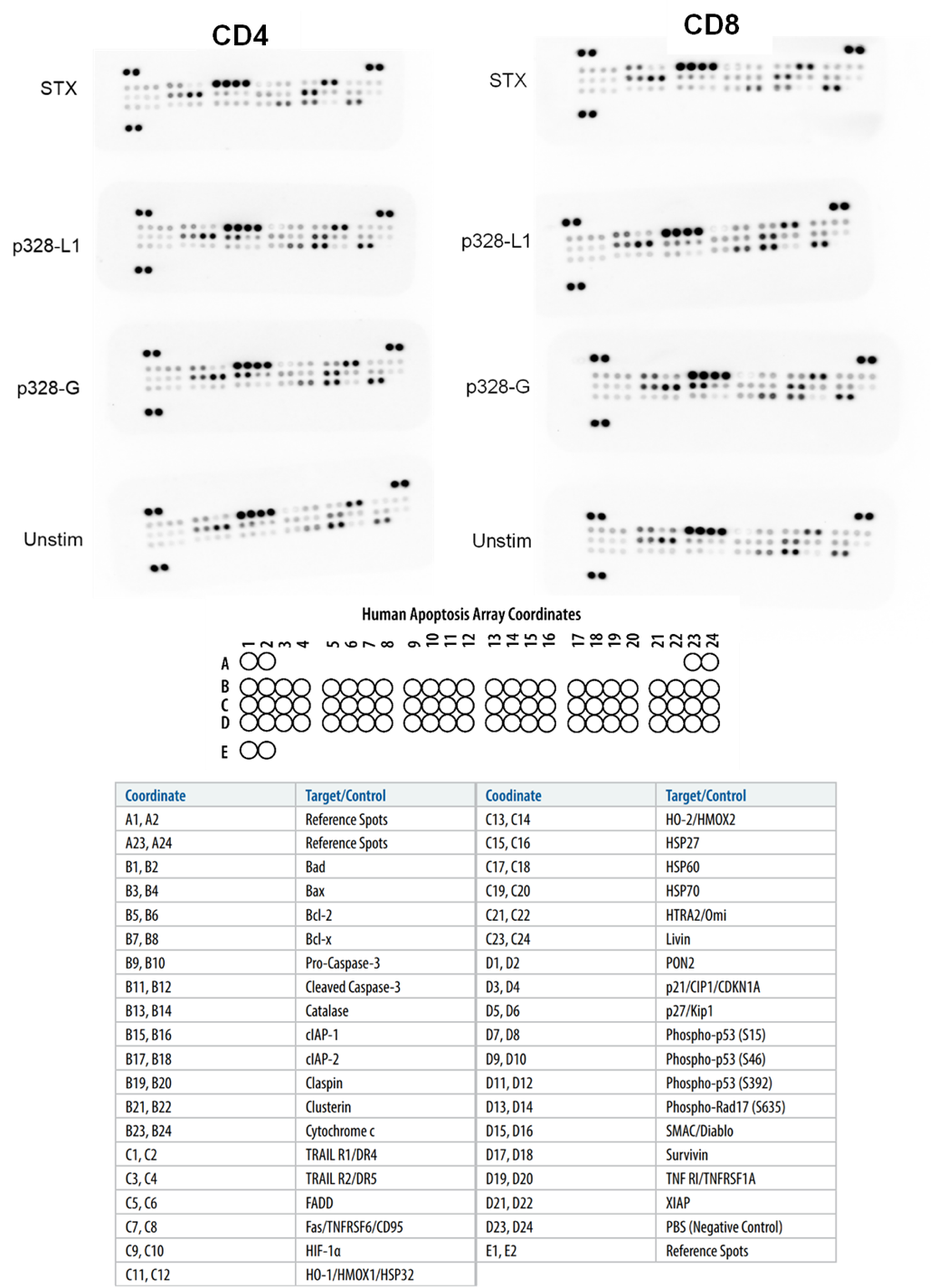

Supplemental Figure 5. Apoptotic protein array from CD4+ and CD8+ T cells.

Complete apoptotic protein array readouts from lysates generated from pooled CD4+ and CD8+ T cells stimulated with p328-IgG vs. p328-L1 beads, relative to unstimulated or STS-treated cells. Pooled lysates comprise cells from 4 separate donors for both cell types; signals were normalized using the dark spots in the corners of the arrays. Protein identities based on array coordinates are provided in the accompanying key and table.

**Supplemental Table 1. Restimulation Bead Formulations**

| Bead Category | Bead Name | Protein Composition | Protein Ratio |
| --- | --- | --- | --- |
| Activation bead | **p328** | anti-CD3:anti-CD28 | 1:1 |
| Restimulation beads with anti-CD28 | **p328-IgG** | anti-CD3:anti-CD28:IgG1κ | 1:1:1 |
|  | **p328-L1** | anti-CD3:anti-CD28:PD-L1 | 1:1:1 |
| PD-L1 titration beads | **pL1_50** (same as **p328-L1**) | anti-CD3:anti-CD28:IgG1κ:PD-L1 | 1:1:0:1 |
|  | **pL1_10** | anti-CD3:anti-CD28:IgG1κ:PD-L1 | 1:1:0.8:0.2 |
|  | **pL1_5** | anti-CD3:anti-CD28:IgG1κ:PD-L1 | 1:1:0.9:0.1 |
|  | **pL1_.5** | anti-CD3:anti-CD28:IgG1κ:PD-L1 | 1:1:0.99:0.01 |
|  | **pL1_.05** | anti-CD3:anti-CD28:IgG1κ:PD-L1 | 1:1:0.999:.001 |
|  | **pL1_0** (same as **pIgG**) | anti-CD3:anti-CD28:IgG1κ:PD-L1 | 1:1.87:1:0 |
| PD-L1 synapse beads | **pGG-G** | IgG1κ | 1 |
|  | **pGG-L1** | IgG1κ:PD-L1 | 2:1 |
| Restimulation beads with anti-CD28 | **p3-IgG** | anti-CD3:IgG1κ | 1:2 |
|  | **p3-L1** | anti-CD3:IgG1κ:PD-L1 | 1:1:1 |

**Supplemental Table 2. Antibodies**

| *Agonist/Antagonist Abs* | | | |
| --- | --- | --- | --- |
| Reagent | Clone | Company | Catalog # |
| Anti-CD3 | OKT3 | Biogems | 05121-20 |
| Anti-CD279 (PD-1) | EH12.2H7 | Biolegend | 329926 |
| Anti-CD279 (PD-1) | Pembrolizumab | Selleck | A2005 |
| Anti-CD274 (PD-L1) | Atezolizumab | BioXCell | SIM0009 |
| Anti-CD178 (Fas-L) | NOK-1 | Biolegend | 306416 |
| IgG1κ |  | BioXCell | BP0297 |
| IgG4 |  | BioXCell | CP148 |
| Anti-CD3 | HIT3α | BD Biosciences | 555336 |
| Anti-CD28 | CD28.2 | BD Biosciences | 555725 |
| *Flow Cytometry Abs* | | | |
| Reagent | Clone | Company | Catalog # |
| Anti-CD69 PE | FN50 | Biolegend | 310906 |
| Anti-CD25 PE | M-A251 | Biolegend | 356104 |
| Anti-CD45RO APC | UCHL1 | Biolegend | 304210 |
| Anti-CD27 PE | L128 | BD Biosciences | 340425 |
| Anti-CD279 (PD-1) APC | EH12.2H7 | Biolegend | 329908 |
| Anti-CD274 (PD-L1) APC | 29E.2A3 | Biolegend | 329708 |
| Anti-CD273 (PD-L2) PE | 24F.10C12 | Biolegend | 329606 |
| Anti-CD28 PE | CD28.2 | Biolegend | 302908 |
| Phospho-p44/42 MAPK (Erk1/2) Alexa Fluor® 647 | 197G2 | Cell Signaling | 13148S |
| Rabbit mAb IgG XP® Isotype Control | DA1E | Cell Signaling | 2985S |
| *Immunoblotting Abs* | | | |
| Reagent | Clone | Company | Catalog # |
| Anti-Phosphotyrosine Antibody, 4G10® Platinum | 4G10 | Sigma | 05-1050X |
| Anti-β-Tubulin Mouse mAb | D3U1W | Cell Signaling | 86298S |
| Anti-phospho-LAT | Tyr220 | Cell Signaling | 3584S |
| Anti-phospho-Zap-70 (Tyr319)/Syk (Tyr352) | 65E4 | Cell Signaling | 2717S |
| Anti-phospho-CD3ζ | Tyr142 | Cell Signaling | 67748S |
| Anti-phospho-p44/42 MAPK (Erk1/2) (Thr202/Tyr204) XP® Rabbit mAb | D13.14.4E | Cell Signaling | 4370S |
| Anti-p44/42 MAPK (Erk1/2) Mouse mAb | L34F12 | Cell Signaling | 4696S |
| Anti-Survivin Rabbit mAb | 71G4B7 | Cell Signaling | 2808S |
| Anti-FasL Rabbit mAb | D1N5E | Cell Signaling | 68405S |
| IRDye® 800CW Goat anti-Rabbit IgG Secondary Antibody | Rabbit IgG | LI-COR | 926-32211 |
| IRDye® 680RD Goat anti-Rabbit IgG Secondary Antibody | Rabbit IgG | LI-COR | 926-68071 |
| IRDye® 800CW Goat anti-Mouse IgG Secondary Antibody | Mouse IgG paraproteins | LI-COR | 926-32210 |
| IRDye® 680RD Goat anti-Mouse IgG Secondary Antibody | Mouse IgG paraproteins | LI-COR | 926-68070 |
| IRDye® 680LT Goat anti-Mouse IgG1-Specific Secondary Antibody | Mouse IgG1 paraproteins | LI-COR | 926-68050 |
| IRDye® 800CW Goat anti-Mouse IgG_2b_-Specific Secondary Antibody | Mouse IgG_2b_ paraproteins | LI-COR | 926-32352 |
| IRDye® 800CW Goat anti-Mouse IgG1-Specific Secondary Antibody | Mouse IgG1 paraproteins | LI-COR | 926-32350 |

**Supplemental Table 3. Chemical, peptides, and recombinant proteins.**

| Reagent | Company | Catalog # |
| --- | --- | --- |
| Dynabeads™ M-450 Epoxy | Thermo Fisher Scientific | 14011 |
| Bovine Serum Albumin | Sigma | A9418-10G |
| Sodium phosphate buffer | Made in house |  |
| Recombinant Human PD-L1/B7-H1 Fc Chimera Protein, CF | Biotechne | 156-B7 |
| Propidium Iodide | Thermo Fisher | P1304MP |
| AnnexinV APC | Biolegend | 640920 |
| AnnexinV Binding Buffer | Biolegend | 422201 |
| Triton™ X-100, PROTEIN GRADE® Detergent, 10% Solution, Sterile-Filtered | Sigma | 648463-50ML |
| Sodium Citrate | Sigma | W302600-1KG-K |
| Rnase A | Sigma | 10109142001 |
| 16% Formaldehyde, Methanol-Free | Cell Signaling | 12606S |
| Protease Inhibitor Cocktail (EDTA-Free, 100X in DMSO) | Bimake | B14001 |
| cOmplete™, Mini Protease Inhibitor Cocktail | Sigma | 11836153001 |
| PhosSTOP™ | Sigma | 4906845001 |
| β-Mercaptoethanol | Sigma | M6250-10ML |
| Staurosporine | Cayman Chemical | 81590 |
| 2x Laemmli Sample Buffer | Bio-Rad | 1610737 |
| Intercept® (PBS) Blocking Buffer | Bio-Rad | 927-70001 |
| 5(6)-Carboxyfluorescein diacetate *N*-succinimidyl ester (CFSE) | Sigma | 21888-25MG-F |
