# Supplementary material for: PD-1 protects expanding human T cells from premature restimulation-induced cell death by modulating TCR and CD28 signaling
Source: Cell Death Dis. 2026 Feb 26;17(1):272. doi: 10.1038/s41419-026-08530-6 (PMC13004874; doi:10.1038/s41419-026-08530-6)
Supplement: Supplementary file 2 — Supplemental Material - Blots [file 41419_2026_8530_MOESM2_ESM.pdf]

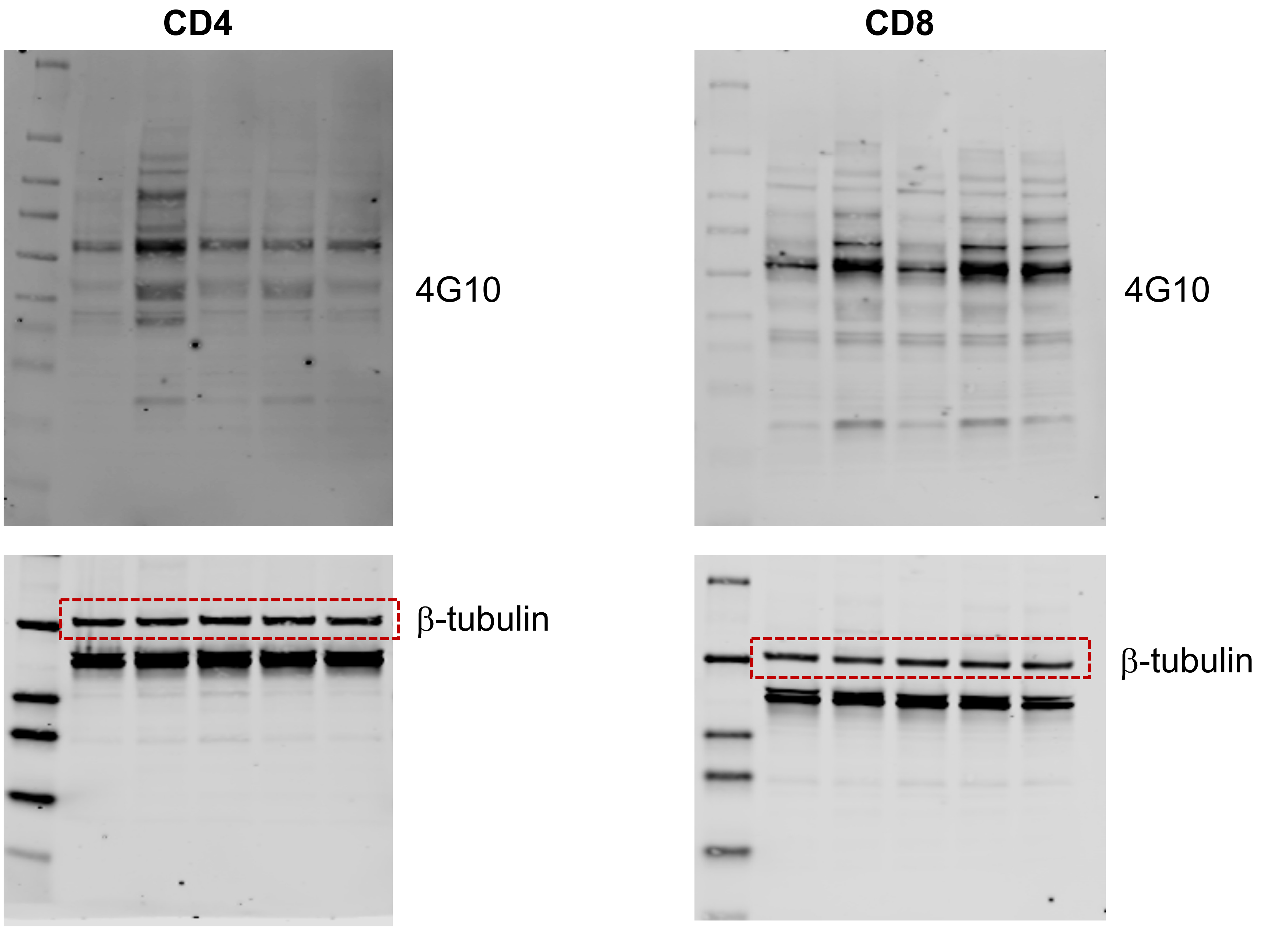

Figure 5A (full length western blots)

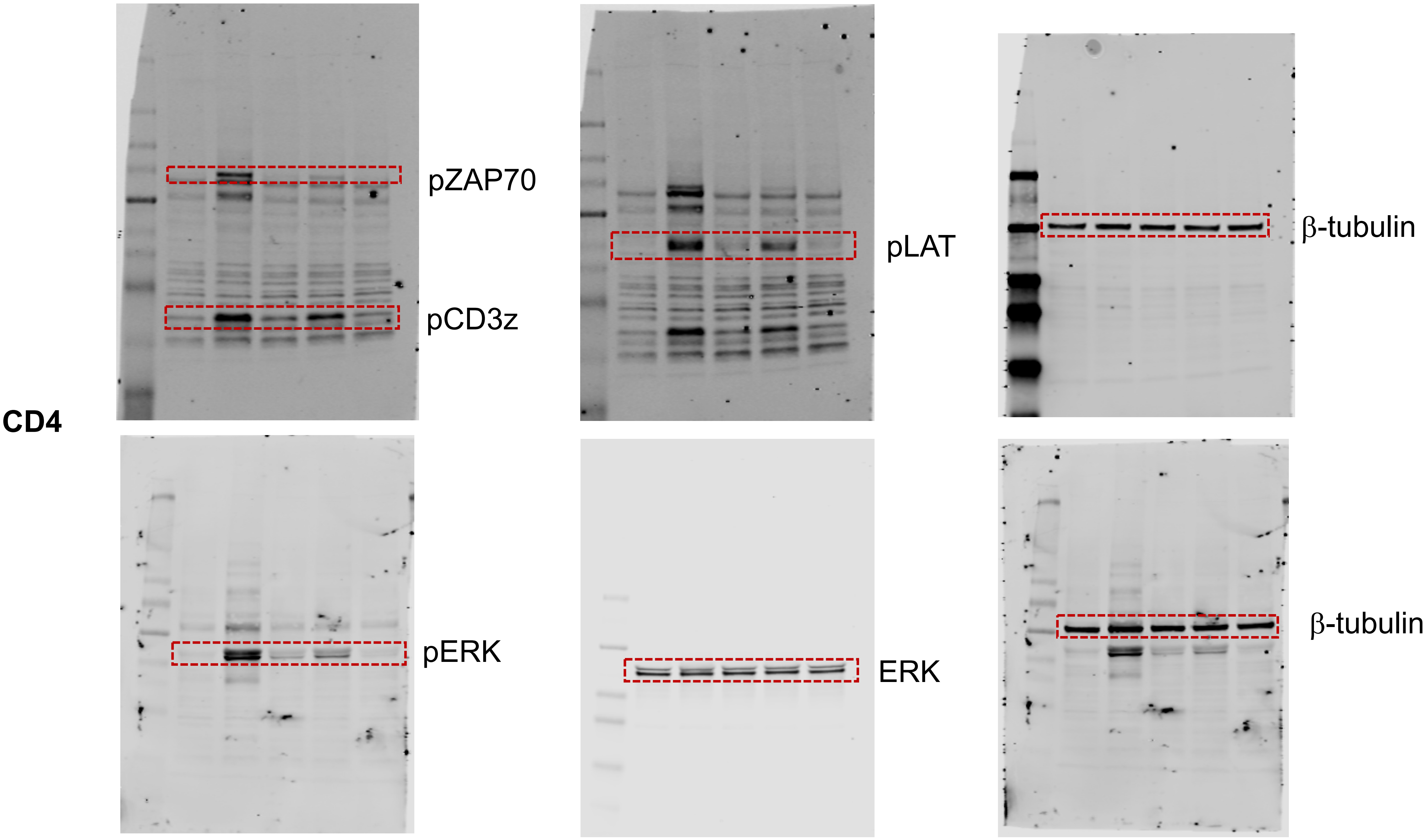

Figure 5B (full length western blots)

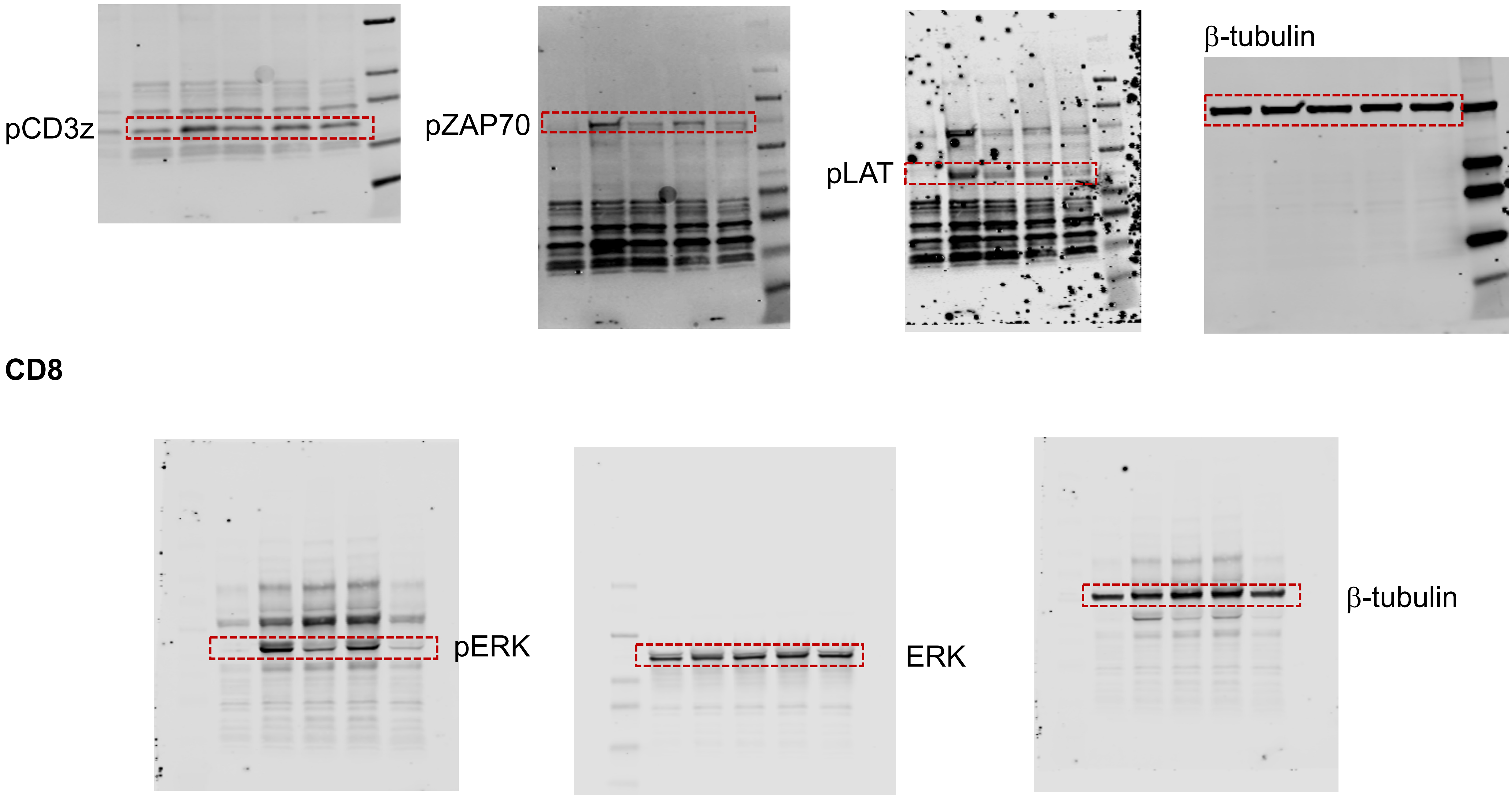

Figure 5B (full length western blots)

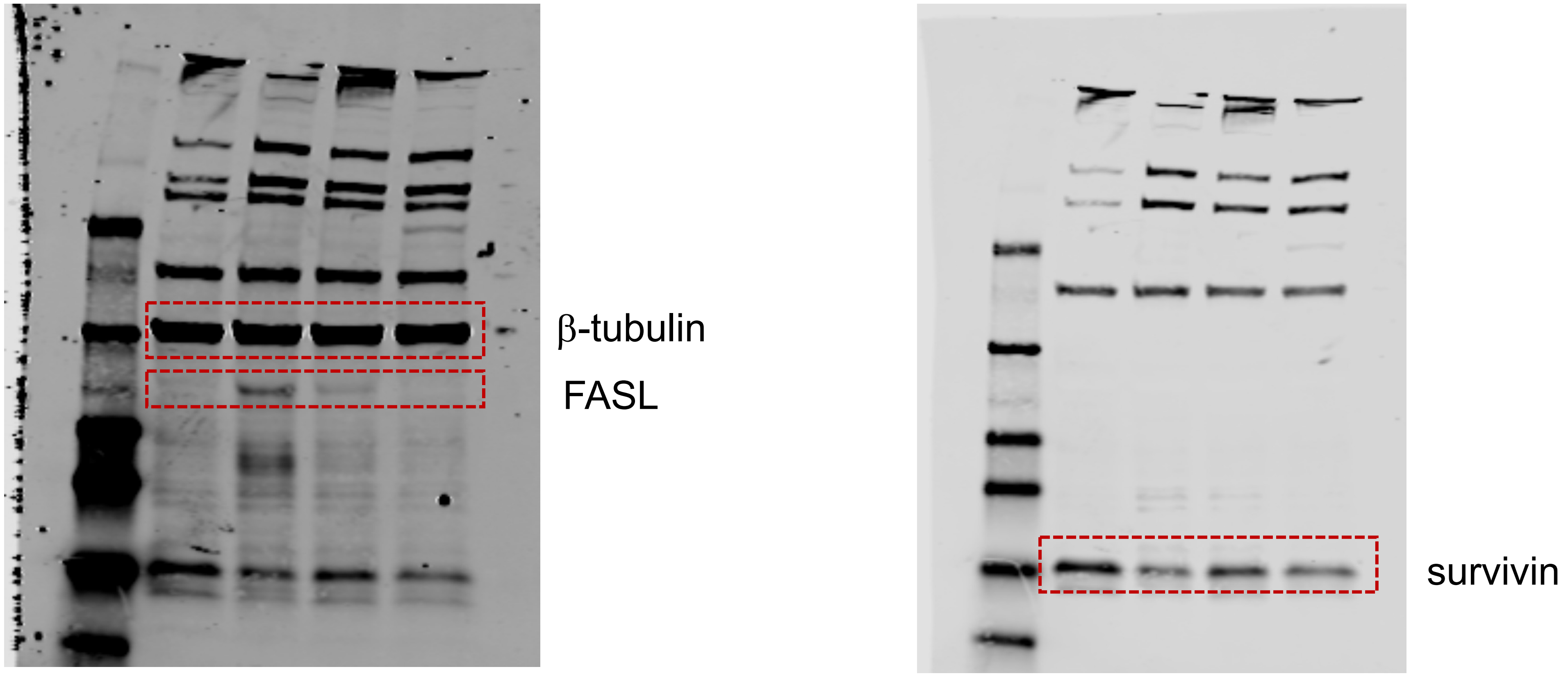

Figure 6B (full length western blots)
